# Association of Sleep Duration and Insomnia Symptoms with Components of Metabolic Syndrome and Inflammation in Middle-Aged and Older Adults with Metabolic Syndrome in Taiwan

**DOI:** 10.3390/nu11081848

**Published:** 2019-08-09

**Authors:** Ahmad Syauqy, Chien-Yeh Hsu, Hsiao-Hsien Rau, Adi Lukas Kurniawan, Jane C-J Chao

**Affiliations:** 1 School of Nutrition and Health Sciences, College of Nutrition, Taipei Medical University, 250 Wu-Hsing Street, Taipei 11031, Taiwan; 2 Department of Nutrition Science, Faculty of Medicine, Diponegoro University, Jl. Prof. H. Soedarto, S.H., Tembalang, Semarang City, Central Java 50275, Indonesia; 3 Department of Information Management, National Taipei University of Nursing and Health Sciences, 365 Ming-Te Road, Peitou District, Taipei 11219, Taiwan; 4 Master Program in Global Health and Development, College of Public Health, Taipei Medical University, 250 Wu-Hsing Street, Taipei 11031, Taiwan; 5 Joint Commission of Taiwan, 31 Sec. 2 Sanmin Road, Banqiao District, New Taipei City 22069, Taiwan; 6 Nutrition Research Center, Taipei Medical University Hospital, 252 Wu-Hsing Street, Taipei 11031, Taiwan

**Keywords:** sleep duration, sleep quality, insomnia symptoms, metabolic syndrome, inflammation, cross-sectional study

## Abstract

The study determined the association of sleep duration and insomnia symptoms with the components of metabolic syndrome and inflammation in middle-aged and older adults with metabolic syndrome in Taiwan. This cross-sectional study used the database compiled in Taiwan between 2004–2013. A total of 26,016 volunteers aged 35 years and above were selected. Metabolic syndrome was defined according to the International Diabetes Federation. Compared with regular sleep duration (6–8 h/day), short (<6 h/day) or long sleep duration (>8 h/day) and insomnia symptoms significantly increased the odds ratios of high waist circumference, high blood pressure, low high-density lipoprotein-cholesterol, high triglycerides, high fasting blood glucose, and high C-reactive protein. Insomnia symptoms did not modify the effects of sleep duration on the components of metabolic syndrome and inflammation. Our study suggests that short or long sleep duration and insomnia symptoms may have an adverse effect on metabolic syndrome and inflammation.

## 1. Introduction

The International Diabetes Federation (IDF) stated that metabolic syndrome is characterized by central obesity, elevated blood pressure, dyslipidemia, and impaired blood glucose [1]. Metabolic syndrome elevated the risks of diabetes, cardiovascular diseases (CVD), and mortality [2,3]. A study revealed that individuals with metabolic syndrome tended to have inflammation and might deteriorate the risks of metabolic diseases and CVD [4]. The prevalence of metabolic syndrome has considerably increased in many countries [5,6,7,8,9]. A Nutrition and Health Survey in Taiwan (NAHSIT) between 1993–1996 and 2005–2008 documented that the prevalence of metabolic syndrome in Taiwan increased almost twofold from 13.6% to 25.5%, respectively [10]. The IDF also declared that central obesity was the underlying factor of metabolic syndrome [1]. The prevalence of central obesity (waist circumference >90 cm in men and >80 cm in women) in Taiwan was 28.3% in men and 28.7% in women [11]. Therefore, the identification of modifiable risk factors may be crucial to prevent the development of metabolic syndrome.

Sleep, one of the most important factors, leads to several health-related issues [12]. Some studies revealed that there was a U-shaped relationship between sleep duration and adverse health outcomes [13,14,15,16]. Short sleep duration had negative effects on metabolic diseases such as obesity [13], hypertension [14], diabetes [15], and escalated mortality [16]. The association had also been shown in long sleep duration [13,14,15,16]. Although previous epidemiological studies reported that short or long sleep duration was related to metabolic syndrome and its components [17,18,19,20,21,22] as well as inflammation [23,24], none of the studies have investigated individuals with metabolic syndrome. Furthermore, insomnia, a public health issue, was related to sleep disorders. The overall prevalence of insomnia ranged from 8% to 40% worldwide [25]. However, the relationship between insomnia and metabolic syndrome remains unclear. Some previous studies demonstrated that insomnia was associated with metabolic syndrome [26,27], but some others did not [28,29]. Moreover, to our knowledge, no study has discussed the association of sleep duration and insomnia symptoms with the components of metabolic syndrome and inflammation using a population with metabolic syndrome. Therefore, further study is required in order to elucidate this association. The objective of the study was to determine the association of sleep duration and insomnia symptoms with the components of metabolic syndrome and an inflammatory marker in Taiwanese middle-aged and older adults with metabolic syndrome.

## 2. Materials and Methods

### 2.1. Data Source and Volunteers

We used a cross-sectional study, and the data were provided by a private health firm in Taiwan (MJ Health Management Institution in Taipei, Taoyuan, Taichung, and Kaohsiung) from 2004 to 2013. The institution collected personal demographic, medical, dietary, and lifestyle information by the self-administered questionnaires and provided medical examination such as anthropometric measurements, image examination, and biochemical tests of blood and urine from people who visited the MJ Health Screening Center in Taipei, Taoyuan, Taichung, and Kaohsiung for a regular health check-up. The questionnaires were available in Mandarin and English versions, and had been validated and standardized previously [30,31]. Details of the questionnaires have been published elsewhere [30,31]. All the volunteers filled in a consent form to allow the use of data without personal identification for research only. Detailed information and volunteers recruitment have been described elsewhere [32]. The committee of Taipei Medical University-Joint Institutional Review Board approved this study (TMU-JIRB N201706051). From the MJ database, 60,769 volunteers aged 35 years and above had metabolic syndrome. Of those, 23,377 volunteers who had chronic diseases such as liver disease, renal disorder, or cancer were excluded. After eliminating those with missing data (*n* = 11,376), the final sample size in our study was 26,016 volunteers.

### 2.2. Definitions of Metabolic Syndrome and Inflammation

Metabolic syndrome was characterized as volunteers with central obesity (waist circumference men ≥90 cm; women ≥80 cm specifically for Asia/Taiwan) with two of the following factors: (1) high blood pressure (systolic blood pressure ≥130 mmHg, diastolic blood pressure ≥85 mmHg, or medication of formerly diagnosed hypertension), (2) low high-density lipoprotein-cholesterol (HDL-C) (men <1.03 mmol/L, women <1.29 mmol/L, or particular medication for dyslipidemia), (3) high triglycerides (≥1.70 mmol/L or particular medication for dyslipidemia), and (4) high fasting blood glucose (FBG) (≥5.60 mmol/L or formerly diagnosed type 2 diabetes) [1]. Inflammation was defined as high C-reactive protein (CRP) (≥28.6 nmol/L) [33].

### 2.3. Data Collection

When visiting the MJ Health Screening Center for health check-up, volunteers recorded their sleep condition using a self-administered questionnaire. Volunteers filled the question of “How many hours do you usually sleep a day?” by one the following response options: <4 h, 4–6 h, 6–8 h, or >8 h [31,34]. In this study, we found only a small number of volunteers (*n* = 214, 0.8%) who slept <4 h/day; therefore, we pooled the data with those who slept for 4–6 h/day. To obtain the insomnia symptoms, the volunteers were asked, “How was your sleep condition in the last month?” with the following response options: difficulty initiating sleep, difficulty maintaining sleep, feeling of non-restorative sleep, use of sleeping pills, and sleeping well. According to the American Psychiatric Association [34,35], volunteers who responded to at least one of the former four options mentioned above were considered as having insomnia symptoms, while volunteers who responded that they were sleeping well were considered as not having insomnia symptoms.

Anthropometric and biochemical assessments were done by well-trained professionals at the MJ Health Screening Center and the MJ Central Laboratory, respectively. Volunteers were fasting for 12–14 h prior to blood drawn for biochemical measurements. Weight and height were assessed by an anthropometer (Nakamura KN-5000A, Tokyo, Japan). Body mass index (BMI) was calculated by the formula of kg/m^2^. Waist circumference was assessed at the narrowest between the iliac crest and the bottom of the ribs. Blood pressure was measured by a sphygmomanometer (Citizen CH-5000, Tokyo, Japan) on the right arm. Plasma HDL-C, triglycerides, FBG, and CRP were assessed enzymatically using an auto-analyzer (Hitachi 7150, Tokyo, Japan) [31].

### 2.4. Covariates

We also collected some associated factors of metabolic syndrome including sex, age, marital status, level of education, current drinking and smoking status, physical activity, and diet using the self-administered questionnaire. Sex was dichotomized as men and women. Marital status was defined as never married, married, and divorced. Level of education was categorized as low (high school or below) and high (above high school). Current drinking and smoking status was dichotomized as no and yes. Physical activity was defined as low (<2 h/week) and high (≥2 h/week). Diet was evaluated using a food frequency questionnaire, and pointed to Taiwanese characteristics of dietary patterns as described previously [36,37]. In this study, data on the diet over the past month included milk, dairy products, eggs, meat, seafood, beans, vegetables, fruits, rice, whole grains, root crops, breads, instant noodles, and sugar. Then, we categorized the frequency of consumption into five groups: none or <1 serving/week, 1–3 servings/week, 4–6 servings/week, 1 serving/day, and ≥2 servings/day.

### 2.5. Statistical Analysis

The data are presented as percentages for categorical variables or means ± standard deviations (SD) for continuous variables. For categorical variables, a chi-squared test was performed to investigate the differences in the characteristics of the volunteers across sleep duration. For continuous variables, a general linear model test was conducted for comparison. The multivariable-adjusted logistic regression analysis was used to estimate the association of sleep duration and insomnia symptoms with the components of metabolic syndrome and CRP, and the odds ratios (OR) and 95% confidence intervals (CIs) were determined. We conducted a stratified analysis by insomnia symptoms to evaluate the effect of insomnia symptoms on the association of sleep duration with the components of metabolic syndrome and CRP. All the regression models were controlled for sex (not including waist circumference and HDL-C), age, marital status, level of education, current drinking and smoking status, physical activity, diet, and insomnia symptoms or sleep duration. Volunteers who slept 6–8 h/day and without insomnia symptoms were selected as a reference group. A *p* value <0.05 was considered statistically significant, and SPSS software (version 24, IBM Corp., Armonk, NY, USA) was used in this study.

## 3. Results

Table 1 shows the characteristics of the volunteers across sleep duration. Among 26,016 volunteers (64.9% men and 35.1% women) with metabolic syndrome, 24.0% of volunteers slept <6 h/day, 66.7% slept 6–8 h/day, and 9.3% slept >8 h/day. More than half of the volunteers (*n* = 14,261, 54.8%) had insomnia symptoms, and 41.9% (*n* = 10,901), 22.1% (*n* = 5,750), 25.1% (*n* = 6,530), 50.3% (*n* = 13,086), and 70.1% (*n* = 18,237) of volunteers had elevated systolic blood pressure, elevated diastolic blood pressure, low HDL-C, high triglycerides, and high FBG, respectively. Volunteers who slept <6 h/day or >8 h/day had significantly higher BMI (27.3 ± 2.6 and 27.2 ± 2.6 kg/m^2^ versus 26.6 ± 2.6 kg/m^2^), waist circumference (89.9 ± 8.1 and 89.3 ± 7.8 cm versus 88.8 ± 8.7 cm), systolic blood pressure (124 ± 41 and 121 ± 36 mmHg versus 119 ± 36 mmHg), diastolic blood pressure (73 ± 21 and 72 ± 23 mmHg versus 71 ± 21 mmHg), plasma triglycerides (2.0 ± 1.4 and 2.0 ± 1.2 mmol/L versus 1.9 ± 1.3 mmol/L), fasting blood glucose (6.9 ± 2.6 and 6.3 ± 1.8 mmol/L versus 6.2 ± 1.7 mmol/L), and CRP (31.9 ± 39.1 and 29.7 ± 36.9 nmol/L versus 27.7 ± 33.0 nmol/L) than those who slept 6–8 h/day. Dietary intake was different among the volunteers with different sleep duration, as shown in Table S1.

The odds ratios of the components of metabolic syndrome and CRP across sleep duration are summarized in Table 2. There was a U-shaped relationship of sleep duration with the components of metabolic syndrome and CRP. Compared with the volunteers who slept 6–8 h/day, those who slept <6 h/day had significantly increased odds ratios of high waist circumference, high blood pressure (except for high diastolic blood pressure in model 3), low HDL-C (except for low HDL-C in men in model 1), high triglycerides, high FBG, and high CRP in all models (*p* < 0.05). Meanwhile, volunteers who slept >8 h/day had significantly increased odds ratios of high systolic blood pressure (only in model 3), low HDL-C in men, high triglycerides, and high FBG in all models (*p* < 0.05).

The odds ratios of the components of metabolic syndrome and CRP across volunteers with insomnia symptoms are presented in Table 3. Compared with the volunteers without insomnia symptoms, those with insomnia symptoms had significantly increased odds ratios of high waist circumference, high blood pressure (except for high systolic blood pressure in model 3), low HDL-C, high triglycerides, high FBG, and high CRP (only in model 1) in all the models (*p* < 0.05).

We further used a stratified analysis to determine whether insomnia symptoms influenced the association of sleep duration with the components of metabolic syndrome and CRP, as shown in Table 4. The odds ratios for the components of metabolic syndrome and CRP also explained a U-shaped association with sleep duration in volunteers with or without insomnia symptoms. Compared with the volunteers who slept 6–8 h/day, those who slept <6 h/day with or without insomnia symptoms had significantly increased odds ratios of high blood pressure, high FBG, and high CRP in all models (*p* < 0.05), and low HDL-C in model 1 (*p* < 0.05). Whereas the volunteers who slept >8 h/day with or without insomnia symptoms had significantly increased odds ratios of high systolic blood pressure and high triglycerides in model 1, as well as high CRP in model 2 (*p* < 0.05). Therefore, the association between sleep duration and metabolic syndrome was not modified by insomnia symptoms.

## 4. Discussion

The results were consistent with a U-shaped curvilinear association between sleep duration and the components of metabolic syndrome and inflammation. Short or long sleep duration and insomnia symptoms had an adverse effect on the components of metabolic syndrome and inflammation. Similarly, the previous epidemiological studies found that the U-shaped associations existed between sleep duration and metabolic diseases including obesity [38], hypertension [14], dyslipidemia [39], and diabetes [40]. In addition, the previous studies showed that short sleep duration was linked to metabolic syndrome [17,18,19,20] and inflammation [23]. Contrarily, few studies had found an association between long sleep duration and metabolic syndrome [17,18,21,22] and inflammation [24]. A meta-analysis study indicated that people who slept less than 6–7 h a day were at a higher risk of metabolic syndrome than those who slept between 7–8 h a day, and those who slept more than 8–9 h a day also showed an increased risk for metabolic syndrome [41]. They observed U-shaped associations between people who slept less than 6–7 h daily or more than 8–9 h daily, and the risk of metabolic syndrome as compared with those who slept between 7–8 h a day [41]. Our study found that after fully adjusting for confounding variables, the relationship between sleep duration and the components of metabolic syndrome and inflammation remained strong, suggesting that sleep duration constantly had an influence on the components of metabolic syndrome and inflammatory marker CRP.

There are several biologically plausible pathways that could potentially explain the association of short or long sleep duration with negative metabolic consequences. Short sleep duration may interfere with the homeostasis of some hormonal systems such as ghrelin and leptin. Short sleep duration or sleep restriction was associated with increased ghrelin and reduced leptin [13,42]. An increase in ghrelin and a decrease in leptin could lead to decreased energy expenditure, impaired glycemic control, increased appetite, and elevated caloric intake [13,42]; furthermore, increased energy consumption might further trigger obesity and the development of metabolic syndrome. Other potential mechanisms are that individuals who slept shorter could increase cytokines such as interleukin (IL)-6 and tumor necrosis factor (TNF)-α [43], elevate cortisol secretion, alter growth hormone metabolism [44], and activate the sympathetic nervous system [44]. These alterations may result in insulin resistance and obesity, which are the components of metabolic syndrome. Moreover, short sleep duration led to low-grade inflammation and elevated stress response in the hypothalamic–pituitary–adrenal axis [45,46], which then resulted in metabolic syndrome and CVD [47]. Contrarily, the mechanisms linked to the association between long sleep duration, metabolic syndrome, and inflammation are not clear yet. A previous study revealed that longer sleep duration (>9 h/day) was correlated with elevated CRP levels in women with type 2 diabetes [24,48]. High levels of pro-inflammatory cytokines were associated with increased sleep duration, and elevated CRP concentrations could promote the development of both metabolic disturbance and CVD [24]. Stranges et al. also found that long sleep duration (>8 h/day) was positively correlated with physical inactivity compared with normal sleep duration (6–8 h/day), probably because individuals who slept longer had less time to do physical activity [48].

This study indicated that insomnia symptoms were significantly associated with the components of metabolic syndrome and CRP. Our study was comparable with other studies that indicated that insomnia symptoms—including difficulty falling asleep, difficulty maintaining sleep, and experiencing unrefreshing sleep—were significantly correlated with metabolic syndrome [26,27]. A meta-analysis of epidemiological studies concluded that depressive symptoms, fatigue, and sleep fragmentation were considered as residual confounding factors, and comorbidity might accelerate the development of metabolic syndrome [49]. An epidemiological study also revealed that metabolic syndrome was closely related to insomnia and other sleep disorders [50]. Moreover, the evidence suggests that individuals with insomnia had increased pro-inflammatory cytokines such as IL-6, nuclear factor-κB, and TNF-α [51,52]. This study showed that the association between sleep duration and metabolic syndrome was not changed by insomnia symptoms after adjusting for confounding variables, indicating that insomnia symptoms consistently affected the components of metabolic syndrome.

The mechanism of the relationship of insomnia symptoms with metabolic syndrome and inflammation is not fully understood yet. The hyperactivity of the hypothalamic–pituitary–adrenal axis responding to insomnia could lead to metabolic syndrome [53]. Additionally, insulin resistance may be induced by sleep disorder, including sleep fragmentation or sleep restriction [54,55], which had a significant impact on the development of metabolic syndrome [56]. Accordingly, prolonged sleep deprivation could regulate the impact of glucose metabolism and further develop metabolic syndrome [57]. Our stratified analysis revealed that insomnia symptoms did not significantly influence the association of sleep duration with the components of metabolic syndrome and CRP as reported in the previous study [34], suggesting that there were no differences in the association of sleep duration with metabolic syndrome and inflammation between the individuals with and without insomnia symptoms.

This study has some important strengths. To the best of our knowledge, this was the first study to discuss the association of sleep duration and insomnia symptoms with the components of metabolic syndrome and inflammatory markers in Taiwanese aged 35 years and over with metabolic syndrome. We used the IDF definition to classify the components of metabolic syndrome, which included central obesity as a key component [1]. The prevalence of metabolic syndrome or central obesity has been escalating among Taiwanese aged 35 years and over [10,11]. Therefore, we used the IDF definition to reflect the most prevalent health issues in Taiwan. Moreover, our study had a large sample size from the population of interest. However, this study had certain limitations. First, similar to most other epidemiological studies, we used a self-reported questionnaire to examine sleep duration instead of an objective measurement of sleep using actigraphic or polysomnographic measurement, which was not practical in a large-scale study. Nonetheless, the previous studies found that both self-reported and objective measurements of sleep quality were correlated with an increased risk of metabolic diseases [58,59]. In addition, we did not evaluate sleep apnea, which might have an effect on the association of sleep duration with metabolic syndrome and inflammation, as the individuals often complained of insomnia symptoms such as difficulties falling asleep and frequent awakenings [60]. However, we adjusted for some confounding variables including sex, age, marital status, level of education, current drinking and smoking status, physical activity, and diet. Finally, the cross-sectional study could not specify causality between the variables. Future studies are needed to clarify the mechanisms underlying the association between sleep duration, insomnia symptoms, metabolic syndrome, and inflammation.

## 5. Conclusions

Our study suggests that short or long sleep duration and insomnia symptoms have an adverse effect on metabolic syndrome and inflammation.

## Figures and Tables

**Table 1:** Characteristics of the volunteers across sleep duration.^1^

Variables	Sleep Duration (Hours/Day)			
	<6 (*n* = 6262)	6–8 (*n* = 17,356)	>8 (*n* = 2398)	*p*-Value
Sex				0.045
Men (%)	64.6	64.8	66.8	
Women (%)	35.4	35.4	33.2	
Age (years)	53.7 ± 11.9	53.5 ± 11.8	53.4 ± 11.3	0.464
Marital status				0.000
Not married	3.7	3.1	2.6	
Married	83.2	84.5	87.4	
Divorced	13.1	12.4	9.9	
Level of education				0.005
Low	54.4	52.1	52.3	
High	45.6	47.9	47.7	
Current drinking status				0.000
No	95.4	96.0	91.5	
Yes	4.6	4.0	8.5	
Current smoking status				0.000
No	96.0	96.7	93.0	
Yes	4.0	3.3	7.0	
Physical activity				0.000
Low	48.4	46.2	41.8	
Moderate	45.5	47.8	52.2	
High	6.1	6.0	6.0	
Insomnia symptoms				0.000
No	50.6	38.6	78.7	
Yes	49.4	61.4	21.3	
Body mass index (kg/m^2^)	27.3 ± 2.6	26.6 ± 2.6	27.2 ±2.6	0.000
Waist circumference (cm)	89.9 ± 8.1	88.8 ± 8.7	89.3 ± 7.8	0.000
Systolic BP (mmHg)	124 ± 41	119 ± 36	121 ± 36	0.000
Diastolic BP (mmHg)	73 ± 21	71 ± 21	72 ± 23	0.001
HDL-C (mmol/L)	1.2 ± 0.4	1.3 ± 0.4	1.3 ± 0.3	0.001
Triglycerides (mmol/L)	2.0 ± 1.4	1.9 ± 1.3	2.0 ± 1.2	0.003
FBG (mmol/L)	6.9 ± 2.6	6.2 ± 1.7	6.3 ± 1.8	0.000
CRP (nmol/L)	31.9 ± 39.1	27.7 ± 33.0	29.7 ± 36.9	0.001

BP: blood pressure, HDL-C: high-density lipoprotein-cholesterol, FBG: fasting blood glucose, CRP: C-reactive protein. ^1^ Data are presented as % for categorical variables and mean ± SD for continuous variables.

**Table 2:** Odds ratios (95% CI) of the components of metabolic syndrome and C-reactive protein across sleep duration.

Variables	Sleep Duration (Hours/Day)			
	<6	6–8	>8	*p*-Trend
High waist circumference (men)				
Model 1 ^1^	1.303 (1.061–1.600)	1	1.040 (0.991–1.091)	0.014
Model 2 ^2^	1.236 (1.005–1.520)	1	1.019 (0.971–1.070)	0.027
Model 3 ^3^	1.246 (0.979–1.513)	1	1.030 (0.981–1.081)	0.044
High waist circumference (women)				
Model 1 ^1^	1.434 (1.130–1.819)	1	1.013 (0.961–1.068)	0.012
Model 2 ^2^	1.444 (1.139–1.833)	1	1.010 (0.958–1.065)	0.009
Model 3 ^3^	1.410 (1.112–1.788)	1	1.009 (0.957–1.065)	0.015
High systolic BP				
Model 1 ^1^	1.228 (1.123–1.342)	1	1.048 (0.986–1.114)	0.000
Model 2 ^2^	1.233 (1.131–1.345)	1	1.058 (0.997–1.123)	0.000
Model 3 ^3^	1.242 (1.134–1.350)	1	1.077 (1.017–1.077)	0.000
High diastolic BP				
Model 1 ^1^	1.326 (1.076–1.578)	1	1.011 (0.896–1.141)	0.031
Model 2 ^2^	1.309 (1.019–1.599)	1	1.008 (0.892–1.139)	0.034
Model 3 ^3^	1.296 (0.993–1.596)	1	1.001 (0.885–1.132)	0.044
Low HDL-C (men)				
Model 1 ^1^	1.120 (0.949–1.321)	1	1.089 (1.046–1.134)	0.000
Model 2 ^2^	1.233 (1.042–1.458)	1	1.073 (1.029–1.118)	0.001
Model 3 ^3^	1.239 (1.047–1.465)	1	1.076 (1.033–1.122)	0.000
Low HDL-C (women)				
Model 1 ^1^	1.106 (1.057–1.156)	1	1.005 (0.839–1.203)	0.000
Model 2 ^2^	1.055 (1.005–1.104)	1	1.026 (0.855–1.231)	0.044
Model 3 ^3^	1.061 (1.013–1.111)	1	1.035 (0.862–1.242)	0.042
High triglycerides				
Model 1 ^1^	1.180 (1.047–1.129)	1	1.096 (1.064–1.129)	0.000
Model 2 ^2^	1.188 (1.052–1.341)	1	1.044 (1.013–1.076)	0.002
Model 3 ^3^	1.202 (1.065–1.358)	1	1.048 (1.016–1.081)	0.001
High FBG				
Model 1 ^1^	1.270 (1.105–1.459)	1	1.044 (1.010–1.078)	0.000
Model 2 ^2^	1.316 (1.146–1.511)	1	1.041 (1.008–1.075)	0.000
Model 3 ^3^	1.304 (1.136–1.498)	1	1.038 (1.005–1.072)	0.000
High CRP				
Model 1 ^1^	1.167 (1.027–1.325)	1	1.027 (0.994–1.061)	0.029
Model 2 ^2^	1.173 (1.033–1.331)	1	1.029 (0.996–1.063)	0.020
Model 3 ^3^	1.180 (1.039–1.339)	1	1.029 (0.996–1.063)	0.017

BP: blood pressure, HDL-C: high-density lipoprotein-cholesterol, FBG: fasting blood glucose, CRP: C-reactive protein. The odds ratios across categories of sleep duration were compared with the reference group (6–8 h/day). The variables were defined as high waist circumference (mean): ≥95.8 cm for men and ≥85.2 cm for women (because all the volunteers had central obesity (waist circumference ≥90 cm for men and ≥80 cm for women), mean waist circumference was used as the cutoff point), high systolic blood pressure: ≥130 mmHg, high diastolic blood pressure: ≥85 mmHg, low HDL-C: <1.03 mmol/L for men and <1.29 mmol/L for women, high triglycerides: ≥1.70 mmol/L, high FBG: ≥5.60 mmol/L, and high CRP: ≥28.6 nmol/L. ^1^ Unadjusted. ^2^ Adjusted for sex (not including waist circumference and HDL-C), age, marital status, level of education, current drinking and smoking status, physical activity, and diet. ^3^ Adjusted for sex (not for waist circumference and HDL-C), age, marital status, level of education, current drinking and smoking status, physical activity, diet, and insomnia symptoms.

**Table 3:** Odds ratios (95% CI) of the components of metabolic syndrome and C-reactive protein across volunteers with insomnia symptoms. OR: odds ratios.

Variables	Volunteers with Insomnia Symptoms	
	OR (95% CI)	*p*-Trend
High waist circumference (men)		
Model 1 ^1^	1.109 (1.051–1.169)	0.000
Model 2 ^2^	1.081 (1.024–1.141)	0.005
Model 3 ^3^	1.083 (1.025–1.144)	0.005
High waist circumference (women)		
Model 1 ^1^	1.130 (1.040–1.227)	0.004
Model 2 ^2^	1.059 (1.005–1.115)	0.031
Model 3 ^3^	1.077 (1.004–1.155)	0.038
High systolic BP		
Model 1 ^1^	1.066 (1.056–1.075)	0.000
Model 2 ^2^	1.055 (1.003–1.109)	0.036
Model 3 ^3^	1.050 (0.997–1.105)	0.045
High diastolic BP		
Model 1 ^1^	1.055 (1.029–1.081)	0.000
Model 2 ^2^	1.038 (1.012–1.064)	0.003
Model 3 ^3^	1.043 (1.022–1.065)	0.000
Low HDL-C (men)		
Model 1 ^1^	1.092 (1.052–1.132)	0.000
Model 2 ^2^	1.045 (1.006–1.086)	0.023
Model 3 ^3^	1.045 (1.005–1.085)	0.025
Low HDL-C (women)		
Model 1 ^1^	1.106 (1.066–1.147)	0.000
Model 2 ^2^	1.050 (1.012–1.091)	0.011
Model 3 ^3^	1.050 (1.011–1.090)	0.012
High triglycerides		
Model 1 ^1^	1.053 (1.006–1.102)	0.039
Model 2 ^2^	1.057 (1.010–1.106)	0.019
Model 3 ^3^	1.066 (1.048–1.084)	0.000
High FBG		
Model 1 ^1^	1.031 (1.003–1.060)	0.029
Model 2 ^2^	1.035 (1.006–1.065)	0.017
Model 3 ^3^	1.032 (1.003–1.062)	0.030
High CRP		
Model 1 ^1^	1.078 (1.048–1.108)	0.000
Model 2 ^2^	1.007 (0.979–1.037)	0.618
Model 3 ^3^	1.010 (0.981–1.040)	0.497

BP: blood pressure, HDL-C: high-density lipoprotein-cholesterol, FBG: fasting blood glucose, CRP: C-reactive protein. The odds ratios across volunteers with insomnia symptoms were compared with the reference group (volunteers without insomnia symptoms). The variables were defined as high waist circumference (mean): ≥95.8 cm for men and ≥85.2 cm for women (because all the volunteers had central obesity (waist circumference ≥90 cm for men and ≥80 cm for women), mean waist circumference was used as the cutoff point), high systolic blood pressure: ≥130 mmHg, high diastolic blood pressure: ≥85 mmHg, low HDL-C: <1.03 mmol/L for men and <1.29 mmol/L for women, high triglycerides: ≥1.70 mmol/L, high FBG: ≥5.60 mmol/L, and high CRP: ≥28.6 nmol/L. ^1^ Unadjusted. ^2^ Adjusted for sex (not for waist circumference and HDL-C), age, marital status, level of education, current drinking and smoking status, physical activity, and diet. ^3^ Adjusted for sex (not including waist circumference and HDL-C), age, marital status, level of education, current drinking and smoking status, physical activity, diet, and sleep duration.

**Table 4:** Odds ratios (95% CI) of the components of metabolic syndrome and C-reactive protein across volunteers with or without insomnia symptoms.

Variables	Volunteers without Insomnia Symptoms				Volunteers with Insomnia Symptoms			
	Sleep Duration (Hours/Day)				Sleep Duration (Hours/Day)			
	<6	6–8	>8	*p*-Trend	<6	6–8	>8	*p*-Trend
High waist circumference (men)								
Model 1 ^1^	1.212 (0.858–1.568)	1	1.050 (0.973–1.127)	0.043	1.375 (1.041–1.817)	1	1.055 (0.982–1.134)	0.032
Model 2 ^2^	1.182 (0.869–1.608)	1	1.031 (0.966–1.101)	0.397	1.322 (0.999–1.751)	1	1.035 (0.962–1.113)	0.106
High waist circumference (women)								
Model 1 ^1^	1.753 (1.234–2.490)	1	1.042 (0.949–1.144)	0.006	1.440 (1.056–1.964)	1	1.133 (1.063–1.208)	0.000
Model 2 ^2^	1.512 (1.057–2.161)	1	1.004 (0.912–1.105)	0.077	1.350 (0.987–1.847)	1	1.007 (0.943–1.076)	0.171
High systolic BP								
Model 1 ^1^	1.423 (1.157–1.742)	1	1.111 (1.029–1.199)	0.000	1.634 (1.367–1.954)	1	1.096 (1.014–1.184)	0.000
Model 2 ^2^	1.439 (1.167–1.770)	1	1.085 (1.003–1.173)	0.000	1.650 (1.375–1.981)	1	1.070 (0.988–1.158)	0.000
High diastolic BP								
Model 1 ^1^	1.203 (1.071–1.352)	1	1.065 (0.956–1.187)	0.007	1.160 (1.082–1.244)	1	1.102 (1.034–1.174)	0.000
Model 2 ^2^	1.197 (1.065–1.346)	1	1.063 (0.953–1.185)	0.009	1.153 (1.075–1.236)	1	1.102 (1.034–1.175)	0.000
Low HDL-C (men)								
Model 1 ^1^	1.242 (0.858–1.627)	1	1.066 (0.989–1.143)	0.035	1.387 (1.075–1.790)	1	1.006 (0.963–1.052)	0.042
Model 2 ^2^	1.243 (0.931–1.658)	1	1.006 (0.968–1.046)	0.218	1.360 (1.054–1.756)	1	1.005 (0.962–1.051)	0.061
Low HDL-C (women)								
Model 1 ^1^	1.078 (1.021–1.139)	1	1.033 (0.808–1.320)	0.025	1.183 (1.090–1.284)	1	1.013 (0.775–1.325)	0.000
Model 2 ^2^	1.020 (0.965–1.079)	1	1.011 (0.789–1.295)	0.779	1.142 (1.085–1.199)	1	1.067 (0.842–1.292)	0.807
High triglycerides								
Model 1 ^1^	1.256 (1.064–1.483)	1	1.139 (1.086–1.195)	0.000	1.105 (0.929–1.314)	1	1.058 (1.019–1.100)	0.012
Model 2 ^2^	1.364 (1.151–1.617)	1	1.129 (1.075–1.186)	0.000	1.110 (0.931–1.324)	1	1.002 (0.963–1.043)	0.008
High FBG								
Model 1 ^1^	1.322 (1.087–1.607)	1	1.004 (0.966–1.043)	0.000	1.509 (1.247–1.826)	1	1.058 (1.005–1.112)	0.000
Model 2 ^2^	1.321 (1.083–1.611)	1	1.016 (0.975–1.060)	0.021	1.296 (1.067–1.574)	1	1.064 (1.011–1.121)	0.006
High CRP								
Model 1 ^1^	1.371 (1.152–1.632)	1	1.044 (0.991–1.100)	0.001	1.290 (1.082–1.538)	1	1.063 (1.009–1.121)	0.005
Model 2 ^2^	1.267 (1.062–1.512)	1	1.074 (1.019–1.133)	0.004	1.217 (1.012–1.462)	1	1.056 (1.001–1.115)	0.006

BP: blood pressure, HDL-C: high-density lipoprotein-cholesterol, FBG: fasting blood glucose, CRP: C-reactive protein. The odds ratios across sleep duration were compared with the reference group (6–8 h/day). The variables were defined as high waist circumference (mean): ≥95.8 cm for men and ≥85.2 cm for women (because all the volunteers had central obesity (waist circumference ≥90 cm for men and ≥80 cm for women), mean waist circumference was used as the cutoff point), high systolic blood pressure: ≥130 mmHg, high diastolic blood pressure: ≥85 mmHg, low HDL-C: <1.03 mmol/L for men and <1.29 mmol/L for women, high triglycerides: ≥1.70 mmol/L, high FBG: ≥5.60 mmol/L, and high CRP: ≥28.6 nmol/L. ^1^ Unadjusted. ^2^ Adjusted for sex (not for waist circumference and HDL-C), age, marital status, level of education, current drinking and smoking status, physical activity, and diet.

## Supplementary Materials

The following is available online at https://www.mdpi.com/2072-6643/11/8/1848/s1, Table S1. Dietary intake of the volunteers across sleep duration.
