# Supplementary material for: Association of Sleep Duration and Insomnia Symptoms with Components of Metabolic Syndrome and Inflammation in Middle-Aged and Older Adults with Metabolic Syndrome in Taiwan
Source: Nutrients. 2019 Aug 9;11(8):1848. doi: 10.3390/nu11081848 (PMC6723777; doi:10.3390/nu11081848)
Supplement: Supplementary file 1 [file nutrients-11-01848-s001.pdf]

Supplementary Material: Association of sleep duration and insomnia symptoms with components of metabolic syndrome and inflammation in middle-aged and older adults with metabolic syndrome in Taiwan

Table S1. Dietary intake of the subjects across sleep duration [1].

Variables	Sleep Duration (hours/day)			
	**<6 (*n* = 6,223)**	**6-8 (*n* = 17,356)**	**>8 (*n* = 2,398)**	***p*-Value**
Milk				0.000
None or <1 serving/week	56.2	48.4	49.3	
1-3 servings/week	20.5	27.6	25.8	
4-6 servings/week	9.8	9.2	8.9	
1 serving/day	11.8	13.7	14.7	
≥2 servings/day	1.7	1.1	1.3	
Dairy products				0.000
None or <1 serving/week	71.2	66.1	68.7	
1-3 servings/week	21.6	27.7	24.9	
4-6 servings/week	4.1	3.1	2.9	
1 serving/day	2.9	3.0	3.2	
≥2 servings/day	0.2	0.1	0.3	
Eggs				0.000
None or <1 serving/week	17.6	16.8	19.7	
1-3 servings/week	56.8	55.0	55.6	
4-6 servings/week	14.1	17.4	14.5	
1 serving/day	10.2	10.1	9.3	
≥2 servings/day	1.3	0.7	0.9	
Meat				0.000
None or <1 serving/week	11.4	11.2	11.5	
1-3 servings/week	37.3	37.4	41.5	
4-6 servings/week	22.6	25.4	22.7	
1 serving/day	21.4	20.7	18.9	
≥2 servings/day	7.3	5.3	5.4	
Seafood				0.000
None or <1 serving/week	18.3	17.0	18.3	
1-3 servings/week	47.2	48.0	49.3	
4-6 servings/week	22.5	22.3	20.0	
1 serving/day	10.7	11.2	10.9	
≥2 servings/day	1.3	1.5	1.5	
Beans				0.000
None or <1 serving/week	20.9	22.0	22.0	
1-3 servings/week	50.1	51.4	51.4	
4-6 servings/week	19.2	17.6	17.6	
1 serving/day	8.1	7.8	7.8	
≥2 servings/day	1.7	1.2	1.2	
Light-colored vegetables				0.000
None or <1 serving/week	13.7	14.6	14.0	
1-3 servings/week	52.0	57.2	56.9	
4-6 servings/week	24.3	19.7	20.2	
1 serving/day	8.0	6.6	6.8	

≥ 2 servings/day	2.0	1.9	2.1	
Dark-colored vegetables				0.000
None or <1 serving/week	13.4	16.2	15.2	
1-3 servings/week	55.9	57.9	58.3	
4-6 servings/week	23.6	18.6	18.7	
1 serving/day	5.0	5.7	5.9	
≥2 servings/day	2.1	1.6	1.9	
Fruits				0.000
None or <1 serving/week	26.5	25.4	26.7	
1-3 servings/week	55.3	59.2	59.5	
4-6 servings/week	14.4	12.1	11.0	
1 serving/day	2.9	2.6	2.2	
≥2 servings/day	0.9	0.7	0.6	
Rice				0.000
None or <1 serving/week	17.1	15.0	15.2	
1-3 servings/week	40.6	48.2	50.4	
4-6 servings/week	37.9	29.1	27.8	
1 serving/day	3.1	6.4	5.6	
≥2 servings/day	1.3	1.3	1.0	
Whole grains				0.000
None or <1 serving/week	41.9	43.4	42.5	
1-3 servings/week	31.0	36.2	35.6	
4-6 servings/week	10.7	8.2	8.5	
1 serving/day	11.5	8.3	9.5	
≥2 servings/day	4.9	3.9	3.9	
Root crops				0.000
None or <1 serving/week	45.2	50.2	51.4	
1-3 servings/week	40.6	40.3	38.9	
4-6 servings/week	11.1	7.5	7.2	
1 serving/day	2.1	1.6	2.0	
≥2 servings/day	1.0	0.4	0.5	
Breads				0.000
None or <1 serving/week	39.6	38.5	41.1	
1-3 servings/week	40.1	43.5	42.0	
4-6 servings/week	13.4	11.4	10.0	
1 serving/day	6.3	6.3	6.7	
≥2 servings/day	0.6	0.3	0.2	
Instant noodles				0.000
None or <1 serving/week	82.7	84.1	83.3	
1-3 servings/week	15.4	15.0	15.6	
4-6 servings/week	1.4	0.7	0.8	
1 serving/day	0.2	0.1	0.2	
≥2 servings/day	0.3	0.1	0.1	
Sugar				0.000
None or <1 serving/week	50.4	55.9	55.1	
1-3 servings/week	37.8	35.0	34.8	
4-6 servings/week	7.1	5.7	6.0	
1 serving/day	3.8	2.9	3.6	
≥2 servings/day	0.9	0.5	0.5	

[1] Data are presented as %.
